# Scheduling algorithms for data-protection based on security-classification constraints to data-dissemination

**DOI:** 10.7717/peerj-cs.1543

**Published:** 2023-11-01

**Authors:** Mohammad Mahmood Otoom, Mahdi Jemmali, Wael M. Khedr, Akram Y. Sarhan, Imen Achour, Ibrahim Alsaduni, Abdullah Bajahzar, Mohamed Nazih Omri

**Affiliations:** 1 Department of Computer Science and Information, College of Science, Majmaah University, Majmaah, Saudi Arabia; 2 Mars Laboratory, University of Sousse, Sousse, Tunisia; 3 Department of Computer Science, Higher Institute of Computer Science and Mathematics, University of Monastir, Monastir, Tunisia; 4 Department of Mathematics, Faculty of Science, Zagazig University, Zagazig, Egypt; 5 Department of Information Technology, College of Computing and Information Technology at Khulis, University of Jeddah, Jeddah, Saudi Arabia; 6 Department of Electrical Engineering, College of Engineering, Majmaah University, Majmaah, Saudi Arabia

**Keywords:** Cybersecurity, Data-dissemination, Packet-transmission, Algorithms, Big data, Networks

## Abstract

Communication networks have played a vital role in changing people’s life. However, the rapid advancement in digital technologies has presented many drawbacks of the current inter-networking technology. Data leakages severely threaten information privacy and security and can jeopardize individual and public life. This research investigates the creation of a private network model that can decrease the number of data leakages. A two-router private network model is designed. This model uses two routers to manage the classification level of the transmitting network packets. In addition, various algorithmic techniques are proposed. These techniques solve a scheduling problem. This problem is to schedule packets through routers under a security classification level constraint. This constraint is the non-permission of the transmission of two packets that belongs to the same security classification level. These techniques are the dispatching rule and grouping method. The studied problem is an NP-hard. Eight algorithms are proposed to minimize the total transmission time. A comparison between the proposed algorithms and those in the literature is discussed to show the performance of the proposed scheme through experimentation. Four classes of instances are generated. For these classes, the experimental results show that the best-proposed algorithm is the best-classification groups’ algorithm in 89.1% of cases and an average gap of 0.001. In addition, a benchmark of instances is used based on a real dataset. This real dataset shows that the best-proposed algorithm is the best-classification groups’ algorithm in 88.6% of cases and an average gap of less than 0.001.

## Introduction

Most organizations use common countermeasures such as firewalls, antivirus, and antispyware software, intrusion models, virtual private networks (VPNs), and data encryption in transit to tackle the attacks (Li, 2018). However, such methods are not fully effective. Therefore, several methods and research have been conducted to predict attacks to deal with the increased unpredictable number of cyber-attacks. For example, machine learning, time series, Markov models, data mining, and Bayesian networks are popular methods to predict cyber-attacks (Husák et al., 2018).

A data breach is a serious issue that has caused massive damage to enterprises worldwide. According to Dinger & Wade (2019), a firm’s average security breach damage cost is $3.9 million. The size of a breach has been increasing and has become significant for a large firm. For example, a breach at Equifax exceeded $600 million. While the lack of cyber security attack preparation and response is one reason, insufficient data security-related decision-making is another. Implementing advanced security tools and access control mechanisms could minimize the attacks, but they will not be prevented due to human error, weakness, and psychological manipulation (Dinger & Wade, 2019). The ongoing security threat and data breaches show the need to redesign our systems’ data processing and dissemination and incorporate technology-based data security and privacy policy and strategies.

According to the open system’s interconnections into a seven-layered network, the internet protocols have been viewed, in Fuentes-García, Camacho & Maciá-Fernández (2021), as a set of layers or protocol stack described. To provide end-to-end security solutions, several protocols such as IPSec, SSL, and DNSSEC have been proposed in the latter work. With the rapid growth of smart communication wireless devices, the advancement in computer network architectures, and the invention of 5G mobile communications, traditional data management systems cannot handle massive data. Accordingly, to process higher data rates, guarantee lower data latency, and manage big data, newer network architecture design and better algorithms embedded with security and decision-making capabilities are required (Sultan, Ali & Zhang, 2018). Furthermore, to resolve the network data management under constraints problem, a scheduler was used in their scheme (Alquhayz & Jemmali, 2021) to prioritize highly confidential classified packets in the case the transmission of such packets could be uncertain. The authors used a single router under a fixed time-slot interval (Alquhayz & Jemmali, 2021; Jemmali & Alquhayz, 2020b) to experiment with several proposed heuristics and thus prove the practicality of their research. In addition, in Jemmali & Alquhayz (2020a), the authors developed a scheduler to solve the problem of identical routers in the network.

Data are publicly classified into different classes: top-secret, secret, restricted, and public, and others use other terms like regulatory, public, confidential (highly confidential), and internal. Data classification using security policies has been used in many domains military, business, and healthcare (Katarahweire, Bainomugisha & Mughal, 2020). For instance, a secure data and identity multilevel security outsourcing scheme is proposed in Sarhan & Carr (2017), Sarhan (2017) and Sarhan & Lilien (2019).

Multilevel security (MLS) controls the disclosure of data in trusted and unsafe environments. Only authorized individuals can access, modify, or delete data. The current network behavior is static, which makes the network sluggish in unstable network environments (*e.g.*, traffic patterns or topology changes, or link failures)-thus, there is a need for a multilevel security policy that can be adopted under any circumstances (Burke et al., 2022). Ali & Mathew (2022) proposed a blockchain-based IoT network multilevel security architecture that provides multilevel data protection. The scheme uses cipher ChaCha20 and cellular automata to gain more security and randomness. The same authors claimed that their scheme enhances security and protects against all kinds of attacks by providing multiple levels of encryption; however, their scheme is not flexible and cannot minimize the chances of leakages. In Lin & Hsieh (2022), the authors proposed a scheme that securely transmitted data between cloud service and Internet of Vehicles (IoV) devices. Their scheme uses an M-tree-based elliptic curve and digital signature algorithm (ECDSA) to provide key management for multilevel security infrastructure. Achleitner et al. (2020) MLS scheme enforces the flow policy of information among the inter-node within the network to minimize chances for attacks. Their scheme enforces the MLS policy on the software-defined networks SDN switches by moving the job to the controller. Unlike the proposed MLS methods, our scheme MLS policy ensures the absence of transmitting an identical level of secure information simultaneously.

Different scheduling algorithms proposed in Alharbi & Jemmali (2020), Jemmali (2019), Melhim, Jemmali & Alharbi (2018), Jemmali (2021b), Jemmali (2021a), Haouari, Gharbi & Jemmali (2006), Haouari, Hidri & Jemmali (2008) and Hidri & Jemmali (2020) can be extended and applied to the proposed problem.

Several schemes used reputation systems mechanisms to ensure a trusted routing environment. In Li, Lyu & Liu (2004), the authors proposed a trusted-based routing protocol model recommending the trusted routing node to improve security. In Venkataraman et al. (2015) and Tang et al. (2018), authors embed a trust-based mechanism in the routing path for routing path scheduling. Other authors used Blockchain and trusted public key.

Protocols (Abd El-Moghith & Darwish, 2021) create decentralized inter-domain trusted routing systems and use smart contracts (Ramezan & Leung, 2018) to follow a trusted route to the destination. Detection of malicious nodes using reinforcement learning by automatically discovering the packets number transmitted to node’s neighbor nodes was studied in Mayadunna et al. (2017). In Shukla et al. (2022), the authors proposed a scheme to ensure the privacy of the source location to maintain safety time. The scheme selects multiple phantom nodes based on a dynamic routing generation process, adds a randomly directed path, and transmits the packets through different phantom nodes to ensure security (Boneh & Franklin, 2003).

Several algorithms-treated scheduling problem can be served to solve the studied problem (Jemmali, Hidri & Alourani, 2022; Jemmali et al., 2022a; Hmida & Jemmali, 2022; Alquhayz, Jemmali & Otoom, 2020; Jemmali, Otoom & al Fayez, 2020).

Even though there is a strong need for a multilevel secure data dissemination solution in a military-based environment, not enough research and investment in this domain exist despite the need to use such a solution in the current era where collaboration between businesses and governmental organizations becomes necessary. Furthermore, although several access-control mechanisms have been deployed for secure data dissemination (Ferraiolo, Kuhn & Chandramouli, 2003; Goyal et al., 2006), they are not fully practical in dealing with the multilevel protection of classified big data or data streams needed in a military-based environment. Therefore, the current article proposes a model that relies on two router-based architectures to schedule securely and then disseminate conflict-based multilevel packet security in a critical situation. In addition, other research related to the representation of the network traffic is developed in Melhim, Jemmali & Alharbi (2019) and Melhim et al. (2020).

The contributions of this article are summarized in the following achievements:

• Eight proposed algorithms were coded and assessed to solve an NP-hard problem.
• Explanation of the new constraint regarding the two routers problem giving an example.
• The execution time of the proposed algorithms is efficient and the solution can be obtained in 0.001 s.
• The experimental results show that the best algorithm is *BCG* in 89.1%.

The suggested techniques presented in this article can be exploited and operated to be adopted for other scheduling problems. Our approach is important since it provides optimal security due to its multilevel security that minimizes the level of leaked information in case of cybersecurity attacks compared to other approaches. For example, the highly secure dissemination of packets in our approach justifies our important architectural choice of two routers since two packets that belong to the same level of security are prohibited from being transmitted at the same time, and this only can be accomplished in the main time through more than one router. Thus, if an adversary could capture one highly secure packet at one point through one router, it would not be able to capture a second one simultaneously since our architecture promotes transmitting classified packets with multiple levels of security. Additionally, a review of the literature showed the lack of research on this NP-Hard problem, and the first work is presented in Sarhan, Jemmali & Ben Hmida (2021). Finally, we introduced several algorithms that can help reach metaheuristics or an exact solution for the problem in the future.

The rest of the article is structured as follows: the second section is reserved for the problem description. The third section presents the proposed algorithms. The experimental results is presented in the Experimental results section. The conclusion is given in the Conclusion section.

## Problem Description

The description of the presented problem is as follows. The transmission of different files through two routers into a network will be executed under the proposed constraint. This constraint of the classification of the packets where each file has a security classification level denoted by *SC*_*i*_ where $i\mathrm{ = } \left\{ 1,\ldots ,Ns \right\} $ with *Ns* as the number of security classification levels.

Into the network, all files received by the intelligencer (administrator) will be classified into security classification levels. The intelligencer is the person with high priority and is authorized to assign the security classification level to each file. All packets constructing a file will inherit the security classification level from this file (Sarhan & Jemmali, 2023).

The set of all packets is denoted by *PT* and *Np* is the number of all packets. The packet index is denoted by *j*. The security classification level of the packet *j* is denoted by *SC*_*j*_. The transmission time of each packet is denoted by *t*_*j*_. When a packet *j* is transmitted, the cumulative transmission time is denoted by ${C}_{j}^{1}$ and ${C}_{j}^{2}$, through *R*_1_ and *R*_2_, respectively. We denoted by *T*^1^ and *T*^2^ the total transmission time on the router *R*_1_ and *R*_2_, respectively. The maximum time is *T*_*m*_ = max(*T*^1^, *T*^2^). Two packets cannot be transmitted simultaneously through two routers. This problem is proven to be NP-hard in Sarhan, Jemmali & Ben Hmida (2021).

The security classification level of a packet can be fixed as follows:

• *SC*_1_: Very high-security classification level. This level can be assigned to sensitive data classified as national security data or data with high impact.
• *SC*_2_: High-security classification level. This level can be assigned to sensitive data.
• *SC*_3_: Internal security classification level. This level can be assigned to delicate data.
• *SC*_4_: Normal security classification level. This level can be assigned to non-sensitive data but cannot be disclosed to the public.
• *SC*_5_: Low-security classification level. This level can be assigned to reveal data that can be shared with the public.

Proposition 1 *The summation of all transmission time for all packets can be written as given in Eq. (1):*

(1) \begin{eqnarray*}\sum _{j=1}^{j=Np}{t}_{j}={T}^{1}+{T}^{2}\end{eqnarray*}

Proof 1 The set of packets transmitted through the router *R*_1_ is *Ps*_1_. So, the summation of the transmission time of all packets in *Ps*_1_ is given as ∑_*j*∈*Ps*_1__*t*_*j*_ and equal to *T*^1^. Similarly, the set of packets transmitted through the router *R*_2_ is *Ps*_2_. Thus, the summation of the transmission time of all packets in *Ps*_2_ is given as ∑_*j*∈*Ps*_2__*t*_*j*_ and equal to *T*^2^. On the other hand, all packets are transmitted through *R*_1_or *R*_2_, this gives that ${\mathop{\sum }\nolimits }_{j=1}^{j=Np}{t}_{j}={\sum }_{j\in {Ps}_{1}}{t}_{j}+{\sum }_{j\in {Ps}_{2}}{t}_{j}$. Finally, we have Eq. (1).

Example 1 * Let 13 packets be transmitted through two routers under four security classification levels. The transmission time and the security classification levels for these 13 packets are given in*Table 1*.*

The security level for each packet *j* is presented in Table 2. The methods to adopt the security level classifications is based on randomization methods.

**Table 1:** Transmission time and security classification levels for 13 packets for Example 1.

*j*	1	2	3	4	5	6	7	8	9	10	11	12	13
*SC*_*i*_	1	2	3	4	4	4	4	3	2	2	2	4	1
*t*_*j*_	25	1	27	13	10	4	18	13	3	1	11	10	3

**Table 2:** Security levels for each packet *j* for Example 1.

	*j*				
*SC*_1_	1	13			
*SC*_2_	2	9	10	11	
*SC*_3_	3	8			
*SC*_4_	4	5	6	7	12

It is clear to see that packets {2,9,10,11 } belong to the same security classification level *SC*_2_. Consequently, these packets must not be transmitted simultaneously through *R*_1_ and *R*_2_.

**Figure 1:**
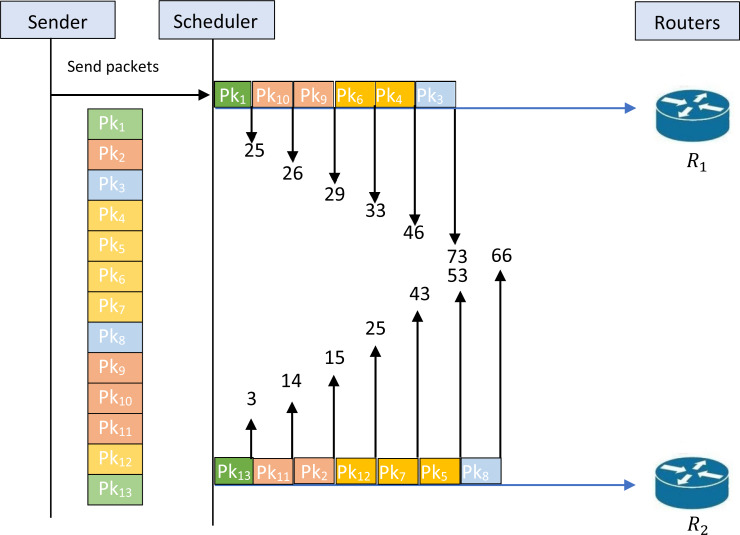
Schedule for an example of an unfeasible solution.

Figure 1 illustrates the schedule for an example of an unfeasible solution. Indeed, the figure shows that the given schedule transmits the packets {1,10,9,6,4,3 } through *R*_1_ and {13,11,2,12,7,5,8} through *R*_2_. The first remarkable infeasibility case is that the packets {1,13} are transmitted simultaneously on the two routers while these packets belong to the same security classification level *SC*_1_ as illustrated in Table 2**.** This infeasibility must not occur on a feasible schedule that can respect the constraint of the security classification levels. Indeed, for each given sequence the assignment on the routers must respect the constraint of the security levels. A test must be made on each schedule to verify the feasibility of the schedule. When the schedule is not feasible a procedure must be called to rectify the schedule and generate a new schedule that respects the proposed constraint.

**Figure 2:**
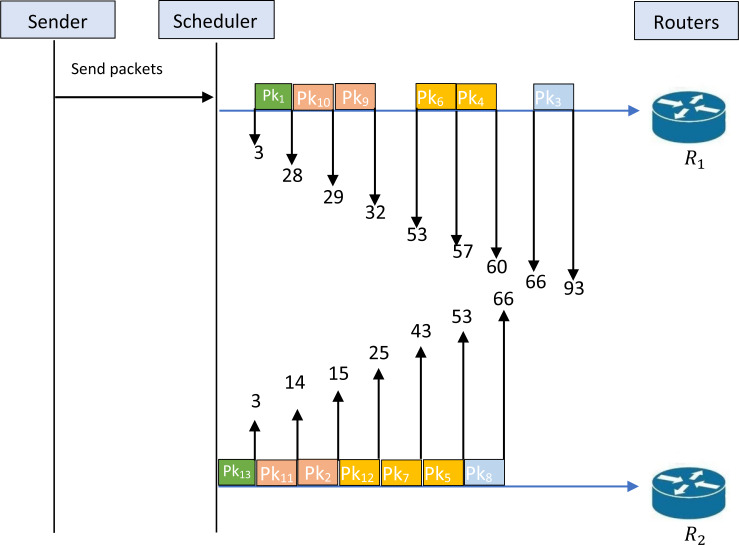
Schedule for an example of feasible solution.

Figure 1 cannot be accepted as a feasible solution to the studied problem; this obligates us to find a feasible solution to the problem that respects the constraint of feasibility. The first intervention that can be made is to separate the transmission of packets {1,13}. Figure 2 illustrates an example of a schedule for a feasible solution. Indeed, this figure shows that the given schedule transmits the packets {1,10,9,6,4,3} through *R*_1_ and {13,11,2,12,7,5,8} through *R*_2_. Now, it is clear that the packets {1,13} are not transmitted simultaneously. Therefore, this schedule is feasible and respects the constraint of security levels. Figure 2 shows that *T*^1^ = 93, while *T*^2^ = 66. So, we have *T*_*m*_ = 93.

## Proposed Algorithms

The proposed algorithms use several techniques to elaborate the results. These techniques are the randomization method, the probabilistic method, the iterative method, and the classification method. Table 3 illustrates the description and abbreviations of the proposed algorithms. The proposed algorithms use several techniques to elaborate the results. These techniques are the dispatching rule method, the grouping method, and the classification method.

### Dynamic decreasing transmission time algorithm (*DTA*)

Firstly, we sort the packets according to the decreasing order of their transmission time. A test will be applied to verify if the security classification level of the packet already transmitted is the same as the selected packet. Suppose the security classification levels are the same. In that case, we select the next packet in the list and test again, and so on until finding the first packet that doesn’t belong to the same security classification level. *TestClassf*(*L*, *X*): determine the security classification level of the packet *X* and find the first packet that doesn’t have the same security classification level of *X* in the list *L*.*Firstpack*(*L*): return the first packet in the list *L*. The procedure *DER*(*PT*) sorts the packets *PT* according to the decreasing order of their transmission time. The procedure  *Sdl*(*PK*) takes in input the packet *PK* and schedules this packet on the router that has the minimum value of *T*^*k*^, with *k* = {1, 2}. The instruction of the algorithm is given in Algorithm 1.

### Grouped security classification level algorithm (*GSC*)

The idea applied to this algorithm is as follows: firstly, we determine a list for each security classification level. The packet decomposes this list in the security classification level *SC*_*i*_ with *i* ∈ {1, …, *Ns*}, and is denoted by *Ls*_*i*_. The packet with index *z* in the list *Ls*_*i*_ will be ${Ls}_{i} \left[ z \right] .$ For each security classification level, sort the packets according to the decreasing order of their transmission time. The group *G*_1_ is constructed by the packets $\{ {Ls}_{i} \left[ 1 \right] ,~~\forall i=\{ 1,..,Ns\} \} $. The group *G*_2_ is constructed by the packets $\{ {Ls}_{i} \left[ 2 \right] ,~~\forall i=\{ 1,..,Ns\} \} $ and so on, until constructed all groups. Now, scheduling the packets belonging to the first group on the two routers. After that, the packets on the second group will be scheduled, and so on, until finishing all groups.

**Table 3:** The description and notation of proposed algorithms.

Algorithm description	Algorithm notation
Dynamic decreasing transmission time algorithm	*DTA*
Grouped security classification level algorithm	*GSC*
Half-shortest classification algorithm	*HSC*
Half-classification decreasing algorithm	*HCD*
Half-classification increasing algorithm	*HCI*
Quarter-classification decreasing algorithm	*QCD*
Quarter-classification decreasing algorithm	*QCI*
Best-classification groups algorithm	*BCG*

Hereinafter, *CGP*() is the function that constructs the groups of packets, and *g* is the number of constructed groups. The instruction of the algorithm is given in Algorithm 2.

### Half-shortest classification algorithm (*HSC*)

Firstly, we sort the packets according to the increasing order of their transmission time. Then, we divide the sorted packets into two groups *G*_1_ and *G*_2_. Two scheduling modalities are applied. The first one is to schedule the packets in *G*_1_ and after that the packets in *G*_2_. The maximum transmission time over the two routers is calculated and denoted by ${T}_{m}^{1}$. The second modality is to schedule the packets in *G*_2_, and after that the packets in *G*_1_. The maximum transmission time over the two routers is calculated ad denoted by ${T}_{m}^{2}$. Consequently, ${T}_{m}=\mathrm{min}\mathrm{}({T}_{m}^{1},{T}_{m}^{2})$.

### Half-classification decreasing algorithm (*HCD*)

This algorithm is based on the same idea as *HSC*. The difference is regarding the initial state of the packets. Here, we don’t initially sort the packets. We divide directly without sorting the packets into *G*_1_ and *G*_2_. Now, in each group, we sort the packets according to the decreasing transmission time order. Finally, we apply the two modalities described above to determine *T*_*m*_.

### Half-classification increasing algorithm (*HCI*)

This algorithm is based on the same idea as *HSC*. The difference is regarding the initial state of the packets. Here, we don’t initially sort the packets. In each group, we sort the packets according to the increasing order of their transmission time. Finally, we apply the two modalities described above to determine *T*_*m*_.

### Quarter-classification decreasing algorithm (*QCD*)

This algorithm is based on the same idea as *HCD*. The difference is regarding the division into the two groups. In *HCD*, we construct the groups with the same number of packets each. However, in this algorithm, we build the groups by taking 1/4 of the packets in *G*_1_ and 3/4 of the packets in *G*_2_. This is the first variant of the proposed algorithm. The second variant is to take 3/4 of the packets in *G*_1_ and 1/4 of the packets in *G*_2_. The best solution will be picked.

### Quarter-classification decreasing algorithm (*QCI*)

This algorithm is based on the same idea as *HCI*. The difference is regarding the division into the two groups. In *HCI*, we construct the groups with the same number of packets each. However, in this algorithm, we construct the groups by quarter division as described in *QCD*.

### Best-classification groups algorithm (*BCG*)

This algorithm is based on the minimum value obtained after running the algorithms *QCI*, *QCD*, *HCI*, and *GSC*.

## Experimental Results

In this section, we detail the results after coding all proposed algorithms in C++ over a computer with an i5 processor and 8G memory.

### Metrics

Several indicators are used to measure the performance of algorithms as follows:

• $\overline{V}$ is the minimum value of *T*_*m*_
• *V* is the *T*_*m*_ value given by the studied algorithm.
• *Ptg* is the percentage of instances when $\overline{V}=V$.
• $ga= \frac{V-\overline{V}}{\overline{V}} $ is the gap between the studied algorithm and the best one
• *Aga* is the average of *ga* through a set of instances.
• *Time* is the average running time (in seconds). We put “*” if the running time is less than 0.001 s.

### Generated dataset evaluation

To assess the proposed algorithms, we used 1,500 instances detailed as follows. The number of packets *Np* is in {6,15,30,40,50,80,150,200}. The number of security classification levels *Ns* is in {3,5,7,8}. Table 4 shows the choice of the number of packets and the number of security classification levels.

The transmission time of each packet is generated following five classes of instances. These classes are based on the uniform distribution *U*(, ) (Sarhan & Jemmali, 2023) and the binomial distribution *B*(, ) (Jemmali & Ben Hmida, 2023). These classes are as follows:

• *Class* 1: *U*(1, 15);
• *Class* 2: *U*(10, 20);
• *Class* 3: *U*(15, 30);
• *Class* 4: *B*(1, 25);
• *Class* 5: *B*(1, 30).

Firstly, a comparison between the proposed algorithms is detailed and discussed. After that, a discussion regarding the proposed algorithms faces those proposed in Sarhan, Jemmali & Ben Hmida (2021), Sarhan & Jemmali (2023) and Sarhan (2023).

Table 5 shows the comparison between the proposed algorithms by *Ptg*, *ga*, and *Time*. This table shows that the best algorithm is *BCG* in 89.1% of cases, an average gap of 0.001 and a running time of 0.015 s. The second best algorithm is *GSC* with *Ptg* = 61.4% and *ga* = 0.005.

**Table 4:** Number of packets and number of security classification levels choice.

*Np*	*Ns*
6	3,5
15,30,40,50,80,150,200	3,5,7,8

**Figure 3:**
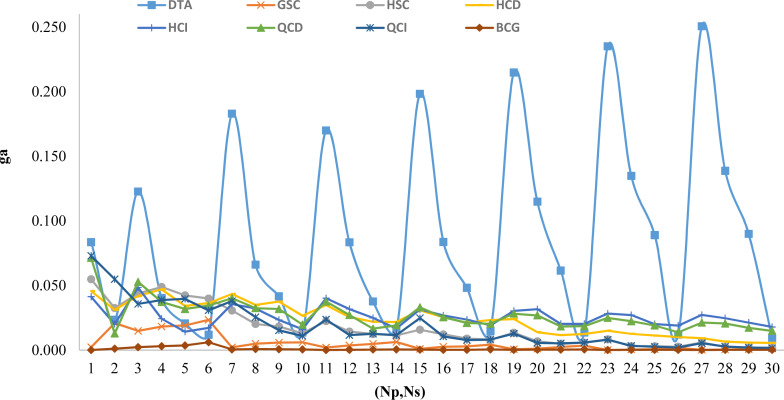
Comparison of all proposed algorithm based on (*Np*, *Ns*) variations.

Based on the generated instances there are 30 tuples of (*Np*, *Ns*). For each tuple, we present the *ga* value for all proposed algorithms in Fig. 3. This figure shows the performance of *BCG* compared to others.

After execution of the algorithms in literature via the used 1,500 instances, a comparison between these algorithms is established. The experimental results show that the best algorithm in Sarhan, Jemmali & Ben Hmida (2021), Sarhan & Jemmali (2023) and Sarhan (2023) are *MDETA*, $\overline{RLT}$, and *RGS*_1_, respectively. The best algorithm from the literature is $\overline{RLT}$. The experimental results show that there is 389 instance where *BCG* is better than $\overline{RLT}$ and 436 instances where $BCG\mathrm{ = }\overline{RLT}$.

**Figure 4:**
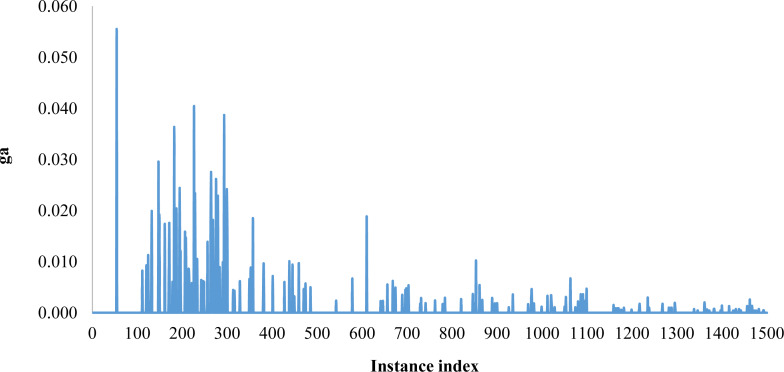
The average gap variation for BCG for each instance among 1,500.

The behavior of the average gap for the algorithm *BCG* for each instance among all the 1,500 ones is illustrated in Fig. 4.

**Table 5:** Comparison between the proposed algorithms by *Ptg*, *ga*, and *Time*.

	*DTA*	*GSC*	*HSC*	*HCD*	*HCI*	*QCD*	*QCI*	*BCG*
*Ptg*	11.8%	61.4%	14.6%	5.5%	15.9%	15.5%	16.4%	89.1%
*ga*	0.088	0.005	0.017	0.024	0.026	0.027	0.018	0.001
*Time*	0.000	0.000	0.005	0.004	0.006	0.004	0.005	0.015

Table 6 illustrates the comparison of *ga* between algorithms according to *Np*. This table shows that the maximum gap value of 0.004 is reached for *BCG* when *Np* = 15. This table shows that the average gap of less than 0.001 is obtained by the *BCG* algorithm when *Np* > 30. The maximum average gap of 0.122 value is obtained when *Np* = 200 by the *DTA* algorithm. While the maximum average gap of 0.004 is obtained by the *BCG* algorithm when *Np* = 15.

**Table 6:** Comparison of *ga* between algorithms according to *Np*.

*Np*	*DTA*	*GSC*	*HSC*	*HCD*	*HCI*	*QCD*	*QCI*	*BCG*
6	0.053	0.012	0.044	0.038	0.031	0.042	0.064	0.001
15	0.049	0.019	0.044	0.040	0.026	0.039	0.036	0.004
30	0.076	0.005	0.020	0.036	0.027	0.031	0.022	0.001
40	0.077	0.004	0.015	0.026	0.028	0.025	0.015	0.000
50	0.086	0.003	0.011	0.025	0.025	0.025	0.013	0.000
80	0.101	0.002	0.008	0.015	0.026	0.023	0.007	0.000
150	0.118	0.001	0.004	0.012	0.024	0.020	0.004	0.000
200	0.122	0.001	0.003	0.007	0.023	0.018	0.003	0.000

Table 7 illustrates the comparison of *ga* between algorithms according to *Ns* for all algorithms. This table shows that the only average gap value of less than 0.001 is obtained by *BCG* when *Ns* = 3. For all values of *Ns* the value of the average is greater or equal to 0.001. On the other hand, the maximum average gap of 0.182 is obtained by the *DTA* algorithm when *Ns* = 3.

**Table 7:** Comparison of *ga* between algorithms according to *Ns*.

*Ns*	*DTA*	*GSC*	*HSC*	*HCD*	*HCI*	*QCD*	*QCI*	*BCG*
3	0.182	0.003	0.024	0.031	0.035	0.039	0.028	0.000
5	0.086	0.007	0.018	0.025	0.027	0.026	0.019	0.001
7	0.055	0.005	0.013	0.020	0.021	0.022	0.012	0.001
8	0.013	0.007	0.012	0.019	0.018	0.020	0.010	0.001

Table 8 illustrates the comparison of *ga* between algorithms according to *Class* for all algorithms. This table shows that the average gap value of 0.001 is obtained by *BCG* independently of the class.

**Table 8:** Comparison of *ga* between algorithms according to *Class*.

*Class*	*DTA*	*GSC*	*HSC*	*HCD*	*HCI*	*QCD*	*QCI*	*BCG*
1	0.085	0.007	0.024	0.032	0.021	0.023	0.030	0.001
2	0.084	0.005	0.014	0.023	0.027	0.026	0.014	0.001
3	0.089	0.005	0.015	0.022	0.027	0.031	0.013	0.001
4	0.088	0.005	0.016	0.021	0.025	0.028	0.017	0.001
5	0.091	0.004	0.016	0.022	0.030	0.028	0.014	0.001

### Benchmark Real-Dataset evaluation

The packet transmission time is estimated based on their size. Indeed, in Pelloso et al. (2018), the authors give a Figure that presented the average transmitted packet size through the network when the time is varying. A correspondence between packet transmission time and the average transmitted packet size can be established easily.

In this subsection, the Real-Dataset used as a benchmark to evaluate the proposed algorithms is the dataset on video packet size and quality performance published in Ukommi (2022). This dataset is composed of two sequence types foreman test video sequence and container test video sequence. The first sequence is divided into two frame numbers. The first number is PSNR (29.11dB) denoted by *PSNR*1. The second number is PSNR (32.20dB) denoted by *PSNR*2. The second sequence is divided into two frame numbers. The first number is PSNR (36.56dB) denoted by *PSNR*3. The second number is PSNR (37.80dB) denoted by *PSNR*4. So, in total there are four different types of performance *PSNR*1, *PSNR*2, *PSNR*3, and *PSNR*4.

The number of packets *Np* is in {10, 15, 20}. The number of security classification levels *Ns* is in {3, 5, 7}. When *Np* = 10, 30 instances are selected for each type of performance and for each number of security classification levels. When *Np* = 15, 20 instances are selected for each type of performance and for each number of security classification levels. When *Np* = 20, 15 instances are selected for each type of performance and for each number of security classification levels. Consequently, the total number of instances of this benchmark is (30 + 20 + 15) × 3 × 4 = 780. Hereafter, the discussion is regarding these 780 instances.

Table 9 shows the comparison between the proposed algorithms by *Ptg*, *ga*, and *Time*. This table shows that the best algorithm is *BCG* in 88.6% of cases, with an average gap of less than 0.001 and a running time of 0.010 s. The second best algorithm is *GSC* with *Ptg* = 64.5% and *ga* = 0.001. As a general conclusion, the results obtained over the five classes in the generated dataset are closer to the results of the real dataset in the benchmark.

**Table 9:** Benchmark comparison of the proposed algorithms by *Ptg*, *ga*, and *Time*.

	*DTA*	*GSC*	*HSC*	*HCD*	*HCI*	*QCD*	*QCI*	*BCG*
*Ptg*	34.4%	64.5%	21.2%	14.9%	20.0%	29.5%	22.1%	88.6%
*ga*	0.052	0.001	0.025	0.043	0.059	0.039	0.017	0.000
*Time*	–	–	0.004	0.004	0.003	0.003	0.003	0.010

After execution of the algorithms in literature via the used 780 instances, a comparison between these algorithms is established. The experimental results show that the best algorithms in Sarhan, Jemmali & Ben Hmida (2021), Sarhan & Jemmali (2023) and Sarhan (2023) are *MDETA*, $\overline{RLT}$, and *RGS*_1_, respectively. The best algorithm from the literature is $\overline{RLT}$. The experimental results show that there is 160 instance where *BCG* is better than $\overline{RLT}$ and 326 instances where $BCG\mathrm{ = }\overline{RLT}$.

The best algorithm proposed in this article as showed in Tables 5 and 9 can be applied to solve the problem with the constraints of window pass studied in Alquhayz & Jemmali (2021), Alquhayz, Jemmali & Otoom (2020), Jemmali, Alharbi & Melhim (2018) and Jemmali et al. (2022b). In addition, the proposed algorithms can be applied and compared to the algorithms proposed in Jemmali, Ben Hmida & Sarhan (2023) Melhim, Jemmali & Alharbi (2018) and Jemmali, Alharbi & Melhim (2018).

## Conclusion

In this article, we investigated the problem of transmitting packets through two routers in the presence of a new constraint that can enhance the security of a network. This constraint is to not allow the transmission of two packets that belong to the same security classification level simultaneously through the two routers. This problem is proved to be NP-hard. Eight algorithms are proposed to solve the studied problem. These algorithms are based essentially on iterative, randomization, and classification approaches. A comparison between these algorithms via different metrics is discussed. The experimental results show that the best algorithm is *BCG* in 89.1% of cases, with an average gap of 0.001 and a running time of 0.015 s. In addition, it is shown that there is no dominance between the proposed algorithms. Compared to the best algorithm in the literature $\overline{RLT}$ the results show that there is 389 instance where *BCG* is better than $\overline{RLT}$ and 436 instances where $BCG=\overline{RLT}$. The future vision is based on three axes. The first axe is the enhancement of the proposed algorithms by applying several metaheuristics and using the results given by the proposed algorithms as initial solutions. The second axe is applying the proposed algorithms to other network problems and tests the performance of these algorithms using different metrics. The last axe is to develop an exact solution for the studied problem using a mathematical formulation or solver Cplex.

##  Supplemental Information

10.7717/peerj-cs.1543/supp-1 Supplemental Information 1 Instances and classes for all 1,500 datasets used.

10.7717/peerj-cs.1543/supp-2 Supplemental Information 2 Code algorithm dispatching rule DRV.

## Additional Information and Declarations

### Data Deposition

The following information was supplied regarding data availability: The data is available in the Supplemental Files.

## References

[ref-1] Abd El-Moghith IA, Darwish SM (2021). Towards designing a trusted routing scheme in wireless sensor networks: a new deep blockchain approach. IEEE Access.

[ref-2] Achleitner S, Burke Q, McDaniel P, Jaeger T, La Porta T, Krishnamurthy S (2020). MLSNet: a policy complying multilevel security framework for software defined networking. IEEE Transactions on Network and Service Management.

[ref-3] Alharbi M, Jemmali M (2020). Algorithms for investment project distribution on regions. Computational Intelligence and Neuroscience.

[ref-4] Ali F, Mathew S (2022). An efficient multilevel security architecture for blockchain-based IoT networks using principles of cellular automata. PeerJ Computer Science.

[ref-5] Alquhayz H, Jemmali M (2021). Fixed urgent window pass for a wireless network with user preferences. Wireless Personal Communications.

[ref-6] Alquhayz H, Jemmali M, Otoom MM (2020). Dispatching-rule variants algorithms for used spaces of storage supports. Discrete Dynamics in Nature and Society.

[ref-7] Boneh D, Franklin M (2003). Identity-based encryption from the Weil pairing. SIAM Journal on Computing.

[ref-8] Burke Q, Mehmeti F, George R, Ostrowski K, Jaeger T, La Porta TF, McDaniel P (2022). Enforcing multilevel security policies in unstable networks. IEEE Transactions on Network and Service Management.

[ref-9] Dinger M, Wade JT (2019). The strategic problem of information security and data breaches. The Coastal Business Journal.

[ref-10] Ferraiolo DF, Kuhn DR, Chandramouli R (2003). Role-based access control.

[ref-11] Fuentes-García M, Camacho J, Maciá-Fernández G (2021). Present and future of network security monitoring. IEEE Access.

[ref-12] Goyal V, Pandey O, Sahai A, Waters B (2006). Attribute-based encryption for fine-grained access control of encrypted data.

[ref-13] Haouari M, Gharbi A, Jemmali M (2006). Bounding strategies for scheduling on identical parallel machines.

[ref-14] Haouari M, Hidri L, Jemmali M (2008). Tighter lower bounds via dual feasible functions. PMS 2008.

[ref-15] Hidri L, Jemmali M (2020). Near-optimal solutions and tight lower bounds for the parallel machines scheduling problem with learning effect. RAIRO-Operations Research.

[ref-16] Hmida AB, Jemmali M (2022). Near-optimal solutions for mold constraints on two parallel machines. Studies in Informatics and Control.

[ref-17] Husák M, Komárková J, Bou-Harb E, Čeleda P (2018). Survey of attack projection, prediction, and forecasting in cyber security. IEEE Communications Surveys & Tutorials.

[ref-18] Jemmali M (2019). Budgets balancing algorithms for the projects assignment. International Journal of Advanced Computer Science and Applications.

[ref-19] Jemmali M (2021a). An optimal solution for the budgets assignment problem. RAIRO-Operations Research.

[ref-20] Jemmali M (2021b). Projects distribution algorithms for regional development. ADCAIJ: Advances in Distributed Computing and Artificial Intelligence Journal.

[ref-21] Jemmali M, Alharbi M, Melhim LKB (2018). Intelligent decision-making algorithm for supplier evaluation based on multi-criteria preferences.

[ref-22] Jemmali M, Alquhayz H (2020a). Equity data distribution algorithms on identical routers.

[ref-23] Jemmali M, Alquhayz H (2020b). Time-slots transmission data algorithms into network.

[ref-24] Jemmali M, Bashir AK, Boulila W, Melhim LKB, Jhaveri RH, Ahmad J (2022a). An efficient optimization of battery-drone-based transportation systems for monitoring solar power plant. IEEE Transactions on Intelligent Transportation Systems.

[ref-25] Jemmali M, Ben Hmida A (2023). Quick dispatching-rules-based solution for the two parallel machines problem under mold constraints. Flexible Services and Manufacturing Journal.

[ref-26] Jemmali M, Ben Hmida A, Sarhan AY (2023). A novel two-routers model based on category constraints secure data-dissemination-aware scheduling in next-generation communication networks. Journal of Network and Systems Management.

[ref-27] Jemmali M, Denden M, Boulila W, Srivastava G, Jhaveri RH, Gadekallu TR (2022b). A novel model based on window-pass preferences for data emergency aware scheduling in computer networks. IEEE Transactions on Industrial Informatics.

[ref-28] Jemmali M, Hidri L, Alourani A (2022). Two-stage hybrid flowshop scheduling problem with independent setup times. International Journal of Simulation Modelling.

[ref-29] Jemmali M, Otoom MM, Al Fayez F (2020). Max-min probabilistic algorithms for parallel machines.

[ref-30] Katarahweire M, Bainomugisha E, Mughal KA (2020). Data classification for secure mobile health data collection systems. Development Engineering.

[ref-31] Li J-H (2018). Cyber security meets artificial intelligence: a survey. Frontiers of Information Technology & Electronic Engineering.

[ref-32] Li X, Lyu MR, Liu J (2004). A trust model based routing protocol for secure ad hoc networks.

[ref-33] Lin HY, Hsieh M-Y (2022). A dynamic key management and secure data transfer based on m-tree structure with multi-level security framework for Internet of vehicles. Connection Science.

[ref-34] Mayadunna H, De Silva SL, Wedage I, Pabasara S, Rupasinghe L, Liyanapathirana C, Kesavan K, Nawarathna C, Sampath KK (2017). Improving trusted routing by identifying malicious nodes in a MANET using reinforcement learning.

[ref-35] Melhim LKB, Jemmali M, Alharbi M (2018). Intelligent real-time intervention system applied in smart city.

[ref-36] Melhim LKB, Jemmali M, Alharbi M (2019). Network monitoring enhancement based on mathematical modeling.

[ref-37] Melhim LKB, Jemmali M, AsSadhan B, Alquhayz H (2020). Network traffic reduction and representation. International Journal of Sensor Networks.

[ref-38] Pelloso M, Vergutz A, Santos A, Nogueira M (2018). A self-adaptable system for DDoS attack prediction based on the metastability theory.

[ref-39] Ramezan G, Leung C (2018). A blockchain-based contractual routing protocol for the internet of things using smart contracts. Wireless Communications and Mobile Computing.

[ref-40] Sarhan A (2023). Novel smart multilevel security approach for secure data outsourcing in crisis. PeerJ Computer Science.

[ref-41] Sarhan A, Jemmali M (2023). Novel intelligent architecture and approximate solution for future networks. PLOS ONE.

[ref-42] Sarhan A, Jemmali M, Ben Hmida A (2021). Two routers network architecture and scheduling algorithms under packet category classification constraint.

[ref-43] Sarhan A, Lilien L (2019). An approach to identity management in clouds without trusted third parties.

[ref-44] Sarhan AY (2017). Protecting sensitive data in clouds using active data bundles and agent-based secure multi-party computation. PhD thesis.

[ref-45] Sarhan AY, Carr S (2017). A highly-secure self-protection data scheme in clouds using active data bundles and agent-based secure multi-party computation.

[ref-46] Shukla A, Singh D, Sajwan M, Verma A, Kumar A (2022). A source location privacy preservation scheme in WSN-assisted IoT network by randomized ring and confounding transmission. Wireless Networks.

[ref-47] Sultan K, Ali H, Zhang Z (2018). Big data perspective and challenges in next generation networks. Future Internet.

[ref-48] Tang J, Liu A, Zhao M, Wang T (2018). An aggregate signature based trust routing for data gathering in sensor networks. Security and Communication Networks.

[ref-49] Ukommi U (2022).

[ref-50] Venkataraman R, Moeller S, Krishnamachari B, Rao TR (2015). Trust—based backpressure routing in wireless sensor networks. International Journal of Sensor Networks.

